# Usability of the IDDEAS prototype in child and adolescent mental health services: A qualitative study for clinical decision support system development

**DOI:** 10.3389/fpsyt.2023.1033724

**Published:** 2023-02-23

**Authors:** Carolyn Clausen, Bennett Leventhal, Øystein Nytrø, Roman Koposov, Thomas Brox Røst, Odd Sverre Westbye, Kaban Koochakpour, Thomas Frodl, Line Stien, Norbert Skokauskas

**Affiliations:** ^1^Department of Mental Health, Regional Centre for Child and Youth Mental Health and Child Welfare (RKBU Central Norway), Faculty of Medicine and Health Sciences, Norwegian University of Science and Technology, Trondheim, Norway; ^2^Department of Psychiatry and Behavioral Neuroscience, The University of Chicago, Chicago, IL, United States; ^3^Department of Computer Science, Norwegian University of Science and Technology, Trondheim, Norway; ^4^RKBU Northern Norway, UiT The Arctic University of Norway, Tromsø, Norway; ^5^Department of Child and Adolescent Psychiatry, St. Olav’s University Hospital, Trondheim, Norway; ^6^Department of Psychiatry, Psychotherapy and Psychosomatics, University Hospital RWTH Aachen, Aachen, Germany

**Keywords:** clinical decision support system (CDSS), child and adolescent mental health services (CAMHS), children and adolescents, attention deficit and hyperactivity disorder (ADHD), usability

## Abstract

**Introduction:**

Child and adolescent mental health services (CAMHS) clinical decision support system (CDSS) provides clinicians with real-time support as they assess and treat patients. CDSS can integrate diverse clinical data for identifying child and adolescent mental health needs earlier and more comprehensively. Individualized Digital Decision Assist System (IDDEAS) has the potential to improve quality of care with enhanced efficiency and effectiveness.

**Methods:**

We examined IDDEAS usability and functionality in a prototype for attention deficit hyperactivity disorder (ADHD), using a user-centered design process and qualitative methods with child and adolescent psychiatrists and clinical psychologists. Participants were recruited from Norwegian CAMHS and were randomly assigned patient case vignettes for clinical evaluation, with and without IDDEAS. Semi-structured interviews were conducted as one part of testing the usability of the prototype following a five-question interview guide. All interviews were recorded, transcribed, and analyzed following qualitative content analysis.

**Results:**

Participants were the first 20 individuals from the larger IDDEAS prototype usability study. Seven participants explicitly stated a need for integration with the patient electronic health record system. Three participants commended the step-by-step guidance as potentially helpful for novice clinicians. One participant did not like the aesthetics of the IDDEAS at this stage. All participants were pleased about the display of the patient information along with guidelines and suggested that wider guideline coverage will make IDDEAS much more useful. Overall, participants emphasized the importance of maintaining the clinician as the decision-maker in the clinical process, and the overall potential utility of IDDEAS within Norwegian CAMHS.

**Conclusion:**

Child and adolescent mental health services psychiatrists and psychologists expressed strong support for the IDDEAS clinical decision support system if better integrated in daily workflow. Further usability assessments and identification of additional IDDEAS requirements are necessary. A fully functioning, integrated version of IDDEAS has the potential to be an important support for clinicians in the early identification of risks for youth mental disorders and contribute to improved assessment and treatment of children and adolescents.

## Introduction

Mental health is a key component of overall health. Mental disorders are amongst the most common and debilitating clinical challenges. For example, depression is one of the leading causes of disability worldwide (1). Furthermore, following the first year of the COVID-19 pandemic, the global prevalence of depression and anxiety increased by 25% (1). While all people are susceptible to developing mental health problems, children and teenagers are most vulnerable, with 75% of all life-time mental disorders having their onset in childhood and adolescence (2, 3). In addition, environmental factors are more likely to negatively impact the developing brain, increasing the risk for mental disorders in youth and children (1, 4). Despite this, access to and availability of timely CAMHS is limited (4). Without appropriate early interventions, children and adolescent mental health symptoms can evolve into potentially lifelong mental disorders, yet 70% of those experiencing mental health problems go without receiving appropriate care (1, 4–6). As part of routine health care, children and adolescents should, but rarely do, receive early assessments for risks associated with mental disorders (7, 8). Detecting and managing these risks as early as possible can help to reduce costs of services as well as societal costs, and ultimately, help alleviate the high demand for more complex treatment services (8, 9).

CAMHS expansion requires not only redistributed health budgets to allocate a greater share of funding toward mental health, but also investment in additional technological resources and mental health informatics (1, 4). Telepsychiatry or virtual reality (VR) exposure therapy exercises, for example, have proven to be effective mental health care (10, 11). Other health information technologies (HIT), such as clinical decision support systems (CDSSs), may have even more potential for service enhancement (12, 13). A CDSS is a tool designed to improve healthcare delivery by enhancing precision and timeliness of medical decisions through provision of support based on targeted clinical knowledge and patient health information (14). CDSSs are designed for various specific purposes, such as risk identification, diagnostics, and prescription management support (9, 12, 14). They can be developed to provide support with the use of clinical practice guidelines, as well as employing artificial intelligence (AI) to map aggregated patient health record data, commonly referred to as “big data” (11, 15, 16). Big data analytics and mental health informatics using AI can provide evidence from multiple sources to allow for an aggregation of knowledge, account for multifaceted patient situations, and gain important insights for future approaches to care (16, 17).

Because of the challenge in juxtaposing normative clinical guidelines, with empirical evidence in the form of care patterns, developing a CDSS requires collaborative, multi-disciplinary efforts to ensure a cohesive balance between the technological innovation and the clinical workflow (12, 18, 19). Human computer interaction (HCI) and user-centered design (UCD) methods allow for simulated experimental and observational approaches that provide valuable insight into user workflow and clinician problem-solving needs. This process informs development, based on close collaborations with the end-users throughout innovation and research (13, 20–22).

Clinical decision support systems have found some significant success in general medicine and adult mental health but have yet to be adequately developed and implemented to CAMHS (18, 23, 24). The development of a CDSS for CAMHS faces systematic obstacles, including the lack of coordination amongst services and the limited accessibility of patient health data records used to develop a CDSS for CAMHS (25). While standardized clinical practice guidelines can be easily modeled for inclusion in a CDSS as part of an electronic health record (EHR) platform, providing decision support based on local practice patterns embedded in aggregated patient data can be challenging, as it requires access to hybrid and multi-source clinical data with approval from ethical committees and adherence to data protection regulations (i.e., General Data Protection Regulations-GDPR) alike (11, 14, 16, 20). Despite the challenges, the integration of health data has continued to exhibit potential for improving healthcare services (16).

Continued digital development, utilizing previously collected patient health data, has the potential to provide innovative solutions to acknowledge limitations within health services (4). With the digitalization of health services across specializations, integration of additional information and data from other information systems, could provide clinicians with transparent and holistic insight into a patient’s current needs (14, 16). Exploiting all possibilities of digital solutions within a CDSS, not only limited to patient health information from the EHR system but additionally encompassing digital case notes and hospital information systems, could provide a more efficient way to address the dynamics involved within CAMHS (11).

In Norway, the Individualized Digital Decision Assist System (IDDEAS) will be the first CDSS in CAMHS that uses both “big data” analytics and standardized clinical guidelines. Norwegian CAMHS are facing substantial increasing demand amidst the COVID-19 pandemic, like elsewhere in the world (26, 27). In 2021, almost 65,000 Norwegian children and adolescents received mental health care – a 14% increase from the previous year (26). Furthermore, over the course of the year nearly 36,000 referrals for mental health care have been reported for children and young people (26). The Norwegian National Association of Child and Adolescent Mental Health Services (N-BUP), established in 1958, has historically been responsible for providing a basis to connect all CAMHS in Norway and continuing to promote coordination and sharing knowledge amongst CAMHS (28). While N-BUP actively helps to facilitate the dissemination and sharing of important CAMHS information through research and management conferences annually, there is still invaluable CAMHS knowledge that has yet to be utilized- previously collected CAMHS individual patient EHR data (i.e., BUP-data) (29). The previously established EHR system of BUP-data was the first of its kind in Norway to be able to provide data comparisons on an individual patient basis (29). While the EHR system has been replaced, utilizing the knowledge acquired within BUP-data, in combination with standardized clinical practice guidelines, has the potential to provide Norwegian CAMHS with additional support to meet the mental health needs of children and adolescents (30). Upon receiving access to this invaluable resource, with support from N-BUP, and in close collaboration with its’ clinicians, the IDDEAS project is developing and researching a CDSS to provide clinicians in Norwegian CAMHS with real-time decision support, in part by BUP-data, but also with standardized clinical guidelines, including DSM-5 and ICD-10 (11).

The IDDEAS prototype is in the process of formative usability testing, including this qualitative study. This study aimed to understand CAMHS clinicians’ overall perceptions of IDDEAS prototype usability while also examining potential barriers to implementation and specific needs to be met in the development of the CDSS. The objectives of this study were to 1) explore clinicians decision-making processes; 2) investigate the perceived usability and functionality of the IDDEAS prototype; and 3) identify the user-perspectives on IDDEAS, to inform continued development and feasibility within Norwegian CAMHS.

## Materials and methods

### Study design

**FIGURE 1:**
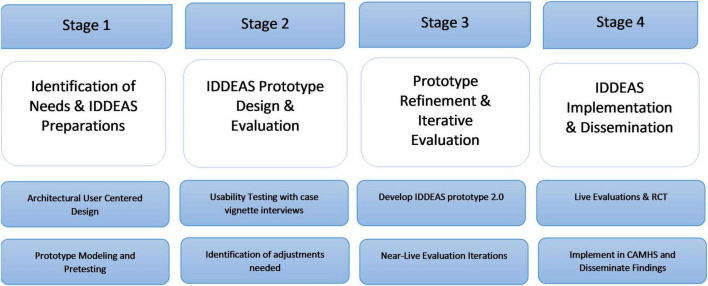
Individualized Digital Decision Assist System (IDDEAS) project study design overview.

This is a mixed-methods study to evaluate IDDEAS, a decision support system for diagnosis and treatment of children and adolescents in Norwegian CAMHS. The IDDEAS project is organized into the following stages: (1) The Assessment of Needs and Preparation of IDDEAS; (2) The Development of the IDDEAS CDSS model; (3) The Evaluation of the IDDEAS CDSS; and (4) Implementation and Dissemination (see Figure 1). This qualitative study reports on the interviews conducted as one component of the usability evaluation of the first IDDEAS prototype (11).

This evaluation process utilizes user-centered design (UCD) methods, with the testing of the CDSS conducted in phases of developmental iterations. The UCD methods include formative usability sessions (12, 31), cognitive walk-through/think-aloud procedures (5, 32), iterative development with end-users, and utilization of both qualitative and quantitative methods of inquiry (31, 33). As part of UCD, the iterative development of the CDSS involves continuous collaboration with CAMHS clinicians. The specific methods and the development plan are detailed in the IDDEAS project protocol (11). The present study serves as the first usability test, using UCD methods to investigate Norwegian CAMHS clinicians’ perceptions of the usability, utility, and overall functionality of the IDDEAS prototype.

### IDDEAS prototype

**FIGURE 2:**
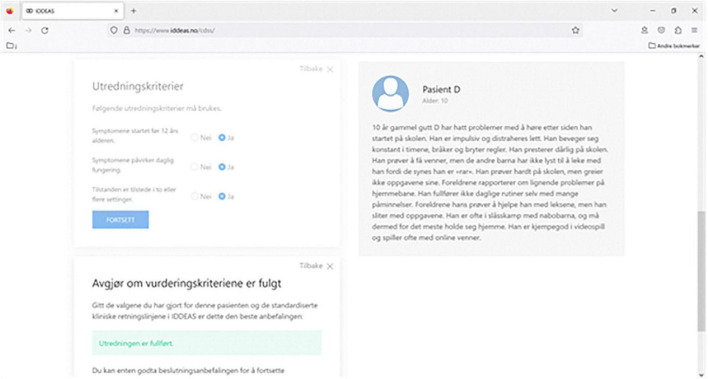
Individualized Digital Decision Assist System prototype software screenshot.

The IDDEAS prototype allows for exploration of the ability of IDDEAS guidelines to provide decision support for diagnosis and treatment of attention deficit and hyperactivity disorder (ADHD) (see Figure 2). ADHD is a neurodevelopmental disorder characterized by inattention, hyperactivity, and impulsivity, ultimately causing impaired functioning for the individual (9). The IDDEAS prototype at this stage uses ADHD as the first clinical model paradigm. Preparation of IDDEAS includes the validation of the clinical materials and the user-interface. The IDDEAS guidelines were previously validated by the IDDEAS clinical research team using the DSM-5 and ICD-10 criteria. Focus groups were used to pre-test content prior to the IDDEAS prototype evaluation.

Each IDDEAS prototype evaluation session included having a clinician participant complete a concurrent, cognitive walk through/think-aloud procedure, as they critically appraised hypothetical patient case scenarios developed from real cases within CAMHS. A total of 20 patient case scenarios were collaboratively designed and validated by the IDDEAS team (BL, NS, RK). Out of the 20 possible cases, each participant was randomly assigned four to assess, two of which were to be assessed while using the IDDEAS prototype (ADHD modeled guidelines) and two without. Use of IDDEAS was similarly randomly assigned. Throughout the assessment of the four cases, participants were asked to follow a think-aloud procedure and provide a concurrent walk through of the clinical procedure they would follow if the patients were real. They were also asked to provide additional patient information they perceived to be potentially necessary to complete their clinical assessment. Finally, participants were asked to provide their overall perceptions of the IDDEAS prototype and its usability, functionality, and potential utility.

### Setting and sampling

The participants (*n* = 20) were those who first participated from the larger cohort evaluation of the IDDEAS prototype (11). We (CC) directly contacted all potential participants who had been recommended by N-BUP board members and those from a random list of service providers. To promote privacy and confidentiality, an invitation email with background information about the IDDEAS team and consortium, as well as the project’s scope and aims, was sent to each potential participant. We (CC) met with each participant prior to the evaluation session in order to go through the proposed study procedure, as well as to provide participants with an opportunity to get acquainted and ask any potential remaining questions. Upon agreeing to participate in the study, each participant created their own profile on the IDDEAS portal and in accordance with the Norwegian Centre for Research Data (NSD) protocol, completed the informed consent process. Initial focus group discussions and pre-testing sessions were conducted beginning in March 2020, with the interviews taking place until Spring 2022.

### Research instrument

A semi-structured interview guide with five questions was developed collaboratively by the IDDEAS team, based on the specific research question and the overall objectives of the IDDEAS project. The interview guide was created following the Mayring qualitative content analysis (QCA) approach (34) and is similar to those implemented by Schaaf et al. (12) and Baysari et al. (30). The final interview guide was confirmed by the IDDEAS team and translated, making it available in both English and Norwegian (see Supplementary Appendix 1). We (CC) conducted preliminary internal testing with members of the IDDEAS team. After the internal testing, a small focus group interview was held with four Norwegian CAMHS psychologists and psychiatrists who all met the inclusion criteria for the qualitative study. Participants were deemed eligible for inclusion if they were either a child and adolescent psychiatrist or psychologist. All potential participants who did not meet the inclusion criteria were excluded. Study participants were given the option to choose to complete their interview in English or Norwegian.

### Data collection

The interviews took place at the end of the IDDEAS prototype usability evaluation sessions. The study was conducted following UCD methodology and standardized criteria for qualitative research, including the consolidated criteria for reporting qualitative research (COREQ) and the Standards for Reporting Qualitative Research (SRQR) (35). The first author (CC) was responsible for interviewing the participants. The research question, interview guide, and qualitative data categorization system were all developed by CC and verified by the IDDEAS team.

After completion of informed consent and establishing a profile with the IDDEAS portal,^1^ participants were invited to meet with CC, either in person or online. Due to COVID-19 meeting regulations and safety requirements, all invitations sent out were *via* the Microsoft Teams online platform. All interviews were recorded and transcribed, word-for-word. All interviews were conducted directly following the completion of the IDDEAS prototype assessment’s case appraisal procedure. All interviews took place within one session and no interviews were repeated or redone. All transcripts were saved within a secure, password protected zip file and stored on the Norwegian University of Science and Technology (NTNU) secure server in preparation for data analysis. No personal or sensitive data was included in accordance with the Norwegian Centre for Research Data (NSD) protocol requirements and research data management permission granted (reference code: 100166).

### Data analysis

**FIGURE 3:**
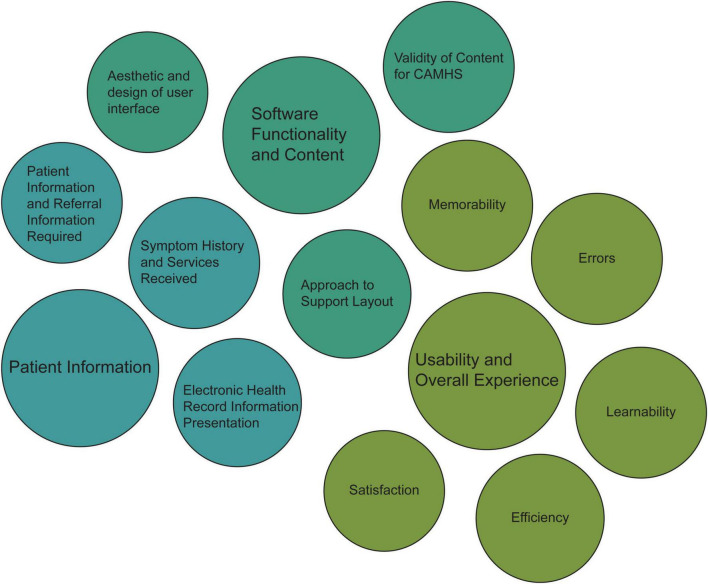
Qualitative content analysis applied main categories and subcategories.

In line with QCA methods, a category system and coding rules were developed for the qualitative data analysis. The system was based on the research question and the study’s objectives, with the specific categories developed to determine which textual passages to take into consideration. Following an inductive category development procedure, the categories are tentative and deduced step-by-step, as applicable. The proposed categories were presented to the IDDEAS team members for theoretical structure verification prior to application to data material and formative/summative checks of reliability. Theoretical based definitions, examples of applicable text passages, and coding rules for each category, were collated within a coding agenda (36). As suggested by Mayring (34, 37), falling within the range of 10–50%, 35% of the transcribed material was checked with the preliminary categories and assessed for adequate representation of theoretical foundation and encompassing the text content. The proposed categories and the coding agenda were presented to the IDDEAS team and underwent revision before completing data analysis. The categories were revised from three main categories and 12 subcategories in Version 1 to a total of 11 subcategories in Version 2 (see Supplementary Appendix 2 for more details). The final category system consisted of three main categories and eleven subcategories (see Figure 3).

All text passages from the interview transcripts were extracted and organized following the deductive category application model (34). The content-analytical coding rules were followed, to keep the process of category application as controlled as possible and to determine the most appropriate category. If there was a text passage that could not be assigned to a category, this was discussed with the IDDEAS team. After assigning all text passages to categories, all included within each category were then summarized and an example quotation was extracted for representation of the content. The extracted quotations that best represented the content of the category were chosen to represent the main findings. Any quotations in Norwegian were translated to English.

## Results

### Participants

The participants represent ten CAMHS clinics. Most participants identified as men (*n* = 11) with the rest identifying as women (*n* = 9). Fourteen were CAMHS psychologists, while 6 were CAMHS psychiatrists. The participants had varied experience working in CAMHS: no participants worked in CAMHS for less than 6 months, one participant had worked in CAMHS for 6–12 months; two participants worked in CAMHS for 1–4 years, and 17 reported to have worked in CAMHS for 4 years or more. The IDDEAS prototype evaluation session duration ranged from 30′20′′ to 93′51′′ with the 5-question interview mean duration of 6′45′′ ranging from 1′15′′ to 11′34′′.

### Main results by category

The following sections present the results organized by the deductive QCA categories. We provide example comments from each category. Three main categories were extracted: (1) Patient information, (2) Software Functionality, (3) Usability and Overall Experience (see Figure 3).

### Category 1: Patient information

#### Patient information and referral information required

Most of the participants reported concern about insufficient patient information available during the evaluation session. Participants who raised this issue acknowledged that they understood that the evaluation procedure was to intentionally include hypothetical patient case scenarios with limited clinical data, as well as the limited ability to engage with the IDDEAS prototype at this stage in its development. One participant noted:

*“[*…*] well, I thought with very limited patient information it makes it a little more difficult. But in real life setting I think it is a valuable clinical tool.”*

Participants shared that insufficient patient information made it difficult to arrive at one diagnosis and indicated a need for more information in order to adequately utilize suggestions. Participants acknowledged they were missing important information, such as what is currently established about the patient, and the ability for the information to be adjusted accordingly to keep up to date. One participant said:

*“I think it could be clearer what is missing and why does the patient not fulfill ADHD criteria so that I can think critically about it and whether there is something that I missed. To have that structured at the end when I’m finished [*…*] because these symptoms are missing, for instance. So, if I am a bit unsure I can think about it.”*

Finally, it was not easy for participants to speculate how it would be to use the system in the future to input their own patient information changes or adapt to changes in patient data between sessions. It was stated that decision support could be very useful when additional referral information is available and, for junior colleagues, guidance on how to find missing information could be beneficial. One participant offered the following suggestion:

*“I guess a good thing would be if it was a patient I knew but then a question I didn’t know and it’d say I have to get this later and then the score would tell me something based on that- where you have the ability to get a score with the “provisional score” or notifying you that you haven’t answered all of them yet.”*

#### Symptom history and services received

All participants found it important to have information about the patient’s previous symptoms, the diagnostic/treatment history, and any previous services. Participants commonly explained that there was often insufficient information available on the family context and the relationship with parents. For the clinicians to feel they can adequately assess the current patient information, there is a need for thorough presentation of patient history and any collaborative services accessed that inform the patient’s assessment. Multiple participants explained the importance of always having multiple hypotheses for patients, without knowing all services received already. More specifically, participants noted that while there may be patient symptom history available, information from additional services involved in the care was not adequately presented. For example:

*“[*…*] while there was a lot of information about the single individual, there was less about the context of the family [*…*] so I feel like having more of the child’s contacts, as student, etc.”*

#### Electronic health record information presentation

All participants reacted positively to the presentation of the patient information directly adjacent to the guideline support. Participants noted the importance of being able to navigate between the guidelines and the patient information seamlessly, and being able to track location in the guideline, while maintaining access to patient information visible on the other side of the screen. One participant explained:

*“If it was something I thought I knew but when I read it again, I don’t actually know it. So, it is very helpful to have those things next to each other and be reminded of specific criteria so you can systematically see where you are at (for the patient). So, I liked that.”*

Participants noted that not only is displaying the patient information and criteria side-by-side advantageous, but it could be potentially important to have the patient EHR data integrated with the decision support in the future. For example:

*“[*…*] if I have a patient, should I then write in all of the symptoms or the case? The intention will be that you will have the EHR and they will be within (integrated)? You might be receiving alerts, etc. I think it would be very good to receive reminders.”*

### Category 2: Software functionality and content

#### Validity of content for CAMHS

Participants’ perceptions of the IDDEAS prototype functionality varied. Some participants had problems interpreting guideline content; they did not like the phrasing and found instructions difficult to understand. Some participants questioned future functionality of IDDEAS with the prototype guidelines requiring the participants to click through all guidelines support materials, regardless of whether they might need to review that specific information or not. For example, one participant stated:

*“[*…*] I think point 2 is obvious, point three as well actually [*…*] and point 4 as well, I guess. The only point that might be helpful is point 1.”*

While this participant was one who seemed hesitant about the need for providing guideline support throughout the clinical process, others spoke highly of the fact that the IDDEAS ADHD guidelines were detailed and encompassed all components of standardized guidelines. Overall, most participants were pleased to be provided with guideline support that matched what they would instinctively do in their current practice and took comfort in knowing it would be available as novice clinicians might need explicit step-by-step guideline support through their assessment. As one clinician explained:

*“[*…*] I see the diagnostic criteria and I am quite fast/or it quickly is matching with my expectations for what this is. So, I would be more concerned or more skeptical if it was a mismatch with my clinical experience or my knowing of what ADHD criteria are. So, it is logic in that sense.”*

With another participant explaining the potential benefits for novice clinicians by stating:

*“I think IDDEAS is a tool that could be very useful, I think. Especially for young clinicians, [who are] not very experienced and having a system that you click and go further and see those symptoms, the criteria are there and then it helps in a decision making.”*

#### Aesthetic and design of user interface

In terms of the aesthetics and design of the IDDEAS prototype, all but one participant found the prototype to be adequate. These participants reported that the text was easy to read, and the guidelines were easy to use. The participant who did not find the aesthetics and design to be adequate noted that the IDDEAS prototype was not aesthetically pleasing due to being too much like a webpage. Most participants emphasized the simplicity as a positive design element. One participant stated:

*“It was neutral. It just felt neutral. And that is alright because I don’t think it needs to be a visually stimulating experience. But it’s good that there are not many distractions, it is good that it quite clear and clean in a way.”*

#### Approach to support layout

Several clinicians reported trouble with the decision tree guideline format which requires “Yes” or “No” responses when criteria are met, or not. “I do not know” option was also voiced, as illustrated by a statement bellow.

*“I missed the “I don’t know” button, but except for that it was really clean.”*

Another participant explained further:

*“[*…*] when I work in a field where the children’s situations are so complicated, I don’t really want to be guided to a yes or no this early in the process. So, there was something there that I didn’t like so much.”*

However, most felt positive about the decision tree as it helped them structure their thought process and identify components of the guideline support that they liked and other aspects that could be changed for the next IDDEAS version. Participants found that the guideline decision support layout, in step-by-step, informative guideline criteria boxes, helped to structure the clinical assessment process:

*“I liked how, or realized that the further I went, it helped me to organize in a sense, instead of just blurting out everything I thought, it was more structural in a sense [*…*]”*

While this participant found the guideline-based support helped structure their efforts, they also noted that this layout could also negatively impact their work, if they were unable to track their progress in applying the guidelines; they feared losing track of progress if they closed one guideline box.

Participants also mentioned a significant concern about their inability to move directly to guideline criteria to allow for investigating differential diagnoses (i.e., investigate inattention criteria met instead of assessing for hyperactivity) rather than going through the entire ADHD guideline following along with the predetermined sequence of the guideline decision support boxes provided, one-at-a-time. Participants were also clear about the need to expand clinical guidelines beyond ADHD in order to address comorbidities and appropriately address symptoms commonly displayed across multiple disorders. This issue also suggested the need to have multiple guidelines and criteria available to allow for navigation from general to specific components of diagnostic criteria. One clinician explained:

*“One thing I would like is access to all of the guidelines, whenever I want. Because what is going on in my mind is several hypotheses at the same time, and that is what I am appointed and educated to do, a differential diagnostic assessment*…”

### Category 3: Usability and overall experience

#### Satisfaction

Most participants indicated that they were not entirely clear about the potential usefulness and helpfulness of limited (to ADHD only) IDDEAS. However, most were hopeful and intrigued by IDDEAS and were interested in its potential even though the prototype had limited utility, as they could not use it in an interaction with “real patient” information.

Despite limitations of the current prototype, it was judged to be easy to use and participants were interested in seeing the ongoing developments. It seemed clear to all that IDDEAS’ usefulness will increase with the expansion of the diagnostic decision tree and the ability to see whether patient symptoms lie along a threshold. A participant stated that they liked the tool because it helped them to structure their thoughts about the diagnosis. One participant reflected on the ease of use and user-friendliness specifically:

*“It was very user friendly, actually. It was very intuitive and very easy [*…*.] you know normally I wouldn’t really think too much about such things and that’s probably a good thing, which means then it was probably fairly easy to move around inside*…*] I think it was decent.”*

#### Learnability

Learnability in this context refers to the ability to learn how to use the IDDEAS prototype. There were mixed thoughts about the “learnability” of IDDEAS. Participants noted that IDDEAS is intuitive, and there is potential for improved ease of use and helpfulness based on the positive degree of learnability. While some reported initial challenges, it did not take too much time to understand how to go through the system. One clinician explained that they would enjoy learning how to interact with the system in the future, over the current approach to clinical care:

*“I think it was very useful. Like I can see myself finding it more fun to do these evaluations, like it reminded me kind of some sort of game or it’s more pleasing to just look up in the EHR platform and papers and ICD manuals and stuff, if you know what I mean.”*

There were some barriers to the learnability of IDDEAS due to the user interface. More specifically, some participants specified that they found it difficult to use and interpret the prompts. One participant explained:

*“[*…*] it was kind of easy to follow where you should be looking, with the exception of the red and green (buttons) [*…*]”*

Another elaborated further, to explain:

*“[*…*] Sometimes it can be like, okay there is a window there and there, and where do I start or what is most important to read first? [*…*] But I know that a lot of people that I work with are maybe kind of “tech hard” so having a very simple button with “start with this” because we have so many things to think about all the time and other distractions.”*

Some participants explained that when there was too much going on within the layout of the interface, it can be challenging. Participants suggested that adjusting the symbols indicating where to click and the wording used in the notifications could make it easier to learn. One stated:

*“There was something that I had to click back and the X symbol, so (indicating) now quitting everything and then nothing back saying “leaving” or things are saved. The wording or the icons need to make it clear that okay I’ve completed this now [*…*]”*

#### Efficiency

The efficiency of using the IDDEAS prototype was discussed both in terms of the current approaches to guideline provision and the potential improvement with developments. Participants found it hard to assess the efficiency of IDDEAS at this time, largely due to the limited capacity of the prototype. Participants discussed that without seeing the entire program, it is difficult to fully appreciate the actual potential for IDDEAS and its contribution to practice efficiency and quality improvement.

One participant noted that they found it difficult to determine IDDEAS to be usable and useful at this stage due to the phrasing of the alerts in guideline boxes causing some delay. For example, understanding the intention of the “decline/accept recommendation” support message provided within the guideline and becoming acquainted with what exactly this was prompting them to do throughout their patient assessment procedure. Additionally noted was the requirement to click through each guideline support box and all criteria included within the ADHD guideline, negatively impacting the efficiency of their clinical procedure. One participant explained:

*“So my mindset is more on speed and efficiency, and this is slow. It is slowing me down [*…*] and this is more like reading a book, so it is maybe actually more efficient to use the real book.”*

#### Memorability

Memorability in this study refers to the ability of the user to remember the task at hand and the components involved in the procedure. Overall, participants spoke positively of the ability to follow the workflow to assess a patient while using the IDDEAS, even though this may be perceived differently from clinician to clinician, particularly based on their experience. One participant explained:

*“I see especially with new psychologists that I have to make them okay with not knowing all the time and to be curious or uncertain and IDDEAS can help with this by widening the focus at the beginning and then narrowing it down as you go.”*

#### Errors

Participants reported at times having encountered errors with the guideline support (i.e., “Not Supported” message displayed upon acceptance of a recommendation) and the navigation buttons (i.e., inability to close one guideline box without exiting entirety). Additionally, participants specifically discussed encountering glitches with the system generating repeat guideline boxes, the inability to access specific criteria when clicking yes in response to prompt suggestion, or falsely notifying the participant that the guideline is over when they have selected to reject the recommendation and continue their assessment. Participants specified that the errors encountered with the prototype made them find it less usable and appropriate at this stage of development. One participant explained in reference to the inability to access more of the guideline upon declining the guideline recommendation:

*“Going into the project, I am probably on the side of being a little bit skeptical already based on the diagnostic system is trending toward categorical systems regarding children’s health and functioning, so I am probably a little bit difficult to convince regarding the usefulness of such a system*…”

On the other hand, another clinician stated simply in reference to a glitch encountered:

*“Okay besides the glitch [repeated guideline support box] if this is refined it could be very interesting tool, absolutely.”*

## Discussion

This study represents the first phase in the development of the IDDEAS CDSS. It is a qualitive study of how CAMHS clinicians perceived the usability of the IDDEAS prototype. As IDDEAS is developed iteratively and in collaboration with the end-users, revisions and adaptations are expected. This qualitative study provides valuable initial information about the usability of IDDEAS, while also identifying needs based on input from potential end-users, CAMHS clinicians.

Our study suggests that the first IDDEAS CDSS prototype needs to be adapted and adjusted to be perceived as usable and helpful. However, more importantly, there is a consensus amongst stakeholders that there is great potential for its usefulness with further development, as well as an eagerness for engagement in helping to inform the future development of the IDDEAS CDSS.

Clinicians were able to use the simulated explorative procedure to evaluate the usability of the IDDEAS prototype, and the potential for useful and helpful future versions of IDDEAS. Our experimental procedures allowed the clinicians to reflect on IDDEAS and suggest what could be better or different. While there was limited patient information and an inability to interact with a fully formed CDSS, the study allowed for us to learn about clinicians’ preferences with respect to what they need and do not need from the CDSS in CAMHS and EHR integration (38). Similarly, other CDSS development studies that have used hypothetical case scenarios found similar limitations dependent upon the state of the CDSS prototype but still identified important takeaways for the systems further development, including close integration of the patient information from the EHR (31). The main consensus elicited from our findings was the importance of quickly being able to identify patient information that is missing at the time of assessment. In this case, clinicians specified that with growing demand for services it is important to be able to efficiently determine whether a patient referral to CAMHS might be rejected or accepted. Furthermore, with global pandemics seemingly becoming a global societal norm, improved timeliness, and overall efficiency of the provision of care within CAMHS could potentially greatly benefit from further incorporation of well tested and validated HIT, such as a CDSS, as long as it is developed in accordance with end user needs.

Based on our findings, a guideline-based decision support system was helpful, but it needs to be able to provide clinicians with customized suggestions as to which clinical guidelines to reference, based on changes in the patient’s health status as well as services previously accessed. Interacting with the guideline-based support provided clinicians an opportunity to reflect on what they feel is lacking in the platforms currently used in CAMHS and speculate how IDDEAS could help to meet these needs in the future. It is also important to acknowledge that the guideline functionality serves as only one component of the overall functionality of the CDSS. The “decision tree” formatting might not optimally serve all clinicians, despite the formatting of the IDDEAS user interface and overall functionality design of the platform that could provide non-linear-based support for dynamic clinical care. In accordance with the need for improved coordination among services involved in CAMHS (4, 25), the IDDEAS project follows the Local Early Appropriate and Precise (LEAP) model (39). Our results reaffirm that as CAMHS in Norway depend on information coming from other services (i.e., educational and psychological counseling service, and the primary care provider), it is important to ensure the available patient information not only covers their current health status but also any previous care received from other social, school and health services (39, 40). This close collaboration provides the opportunity for customized guideline suggestions and availability of information about involved services, while also allowing early identification of risks. Early risk identification is a critical component of CAMHS to prevent the onset of mental health disorders (9).

Individualized Digital Decision Assist System development will continue to work toward full EHR integration, to keep collaborative efforts in CAMHS coordinated and ultimately to help to provide clinicians with accurate, efficient, and early clinical decision support through efficient and early identification of risks and provision of early intervention. With direct integration with the EHR, it will be possible to examine the potential for identifying previously addressed symptoms, while also identifying potential comorbidities by flagging relevant overlaps across multiple guidelines (5). An integrated CDSS potentially provides specific, adapted suggestions relevant to the care of an individual patient, thus allowing the clinician to determine the extent to which they need to review other materials.

Maintaining this autonomy for the clinicians in CAMHS, allowing for them to be the decision-makers, is important for the acceptance and utility of future IDDEAS versions. As found by Kortteisto et al. (22) for the end-users to find a CDSS useful, they need to first trust it. As reported by Sutton et al. (13), diagnostic support based on patient data can be an advantage while also prove potentially harmful if users’ distrust what is provided by the CDSS. Graphical displays of statistics, access to scientific literature, and references to local EHR patient data patterns were all mentioned as examples of potential future design factors that help reassure clinicians of the CDSS trustworthiness while keeping the clinician as the main decision-maker (40).

The inclusion of support based on BUP-data is important to the clinicians as the end-users but is also important to service users (41). Service users in Norway want to be more involved in their care, including understanding the components of services received and sharing their data for the improvement of overall services (41). In general, clinicians want transparent presentations of EHR data that informs the decision support and recommendations provided for all stakeholders involved, making it more likely that stakeholders will trust and use the CDSS (21).

The results suggest that several attributes of the IDDEAS prototype should be addressed in the next version of IDDEAS (see Table 1). For the development of IDDEAS following UCD methods and an overall iterative approach (11, 30), we expect continued identification of usability barriers and limitations to the design of IDDEAS, as seen commonly in other CDSS development studies (25, 38, 42, 43). For example, as similarly found by Baysari et al. (30) or Giordanengo et al. (43), it is important to design the system from the perspective of the end-user and finding overall improved perceptions of the system usability with the well-integrated EHR (44). The formative usability iterations underlying the design of IDDEAS promotes the ability to adjust as needed to meet the local coordinated CAMHS requirements, such as the provision of data-based recommendations from close integration with the patient EHR in the future. While there were several propositions for how to overcome the currently limiting attributes of the IDDEAS prototype, it will be important to also assess the identified barriers of the next prototype and provide a comparison to be able to understand the usability and potential utility of IDDEAS.

**TABLE 1:** Individualized Digital Decision Assist System (IDDEAS) prototype attributes: Perceived strengths and limitations.

Attribute	Perceived strength	Perceived limitation	Proposed development
The “accept recommendation” guideline support box	Shows the clinician that they are still in charge	Can be unclear for some clinicians how recommendation comes about	Show summary confirmation of what the clinician selected and optional box for where recommendation came from
“Criteria not fulfilled” notification guideline support box	Allows for the clinician to see what they know and what they do not know	Can be unclear based on phrasing of information in box	Provide notification for recommendation with evidence optional to access (BUP-data statistics- regional/department examples); simplify wording and appearance to be clear
Structured layout of guideline support boxes	Provides clinician with reminders for the important information to acquire within the assessment steps and following the structured diagnostic process ensuring reliable and standardized diagnostic procedure	Inability to navigate through the guideline outside of the step-by-step structured support boxes and change the order of assessment, when necessary, given the specific patient context	Provide option to click through guideline boxes to see criteria without selecting- ability to access all guideline boxes regardless of recommendation; display score of fulfilled criteria to show current suggestion for diagnosis, maintains control for clinician yet clear support
Visual display and aesthetics of user interface	Simple and minimalistic is good, not distracting	Webpage layout and not intuitive of how to navigate	Keep simple and minimalistic but also modern to help with engagement; provide navigation labels to allow for clinicians to explore how to navigate interface/use support
“Decision tree” style provision of decision support	Helped to organize structure of thought process and to keep track of decision making in line with the diagnostic criteria	Can feel like support is forcing a decision to be made with only yes or no option; does not fully reflect complexity of patient situations in CAMHS	Including a “I do not know” option so marked items will accumulate into box with summary of missing information to be able to efficiently acknowledge what is not known; use the diagnostic tree where you could look at symptoms falling above or below threshold for assessing the diagnosis
Accessibility of specific guideline criteria within guidelines	Can scroll through previously accessed guideline components	Limited guideline capacity to show all components until navigated through tree	Should provide option for going back/forward into the guideline specifics; if missing information should be able to look up where guideline provides support for criteria
Display of guideline support on user- interface next to patient information	Providing guideline support side by side with patient information could improve efficiency of clinical care; more quickly acknowledged what was needed because of accessibility	Decision support is available next to patient information but not connected so cannot interact with guideline and save any previously noted criteria met by patient	Future design of system should be able to have guideline support integrated with the patient to provide alerts relative to patients’ health information; ability to update criteria in between sessions and adjusts decision support provided to improve efficiency and overall coordination of services

### Strengths and limitations

There are multiple strengths and limitations of this study. First, there is a potential for bias. As participants were recruited by convenience sampling, this potentially attracted clinicians who may be already interested in innovation and potentially more comfortable interacting with technological solutions. While the focus of the study was to identify the CAMHS clinicians’ perceptions of the IDDEAS prototype, it was a limitation that patients and their families were not involved as well (9, 25). Furthermore, as this is one component of a larger study within a multiple part project, the generalizability of our findings is limited. However, our sample size is in accordance with qualitative methodology standards and allows for un-saturated qualitative data and provides development information that will be used to design and execute larger scale IDDEAS usability studies. Additionally, the materials used in addition to the IDDEAS prototype (i.e., patient cases) also proved to have relative limitations, potentially deterring from the ability to assess the usability of the system attributes. However, as seen with other CDSS development studies following UCD designs, the patient cases provide a base for early on prototype testing and thus were intentionally designed to illicit important information for future research and help to identify current clinical needs.

Despite these limitations, there were strengths to the study as well. It was a strength to have the opportunity to work closely with the child and adolescent psychiatrists and psychologists who will ultimately be IDDEAS end-users. With the help and support from N-BUP we were able to recruit participants from multiple regions of Norway to help inform our continued development of IDDEAS. Furthermore, as participants were not provided with access to the IDDEAS platform prior to the usability evaluation and interviews, the study provides an authentic overview of the overall perceived usability, and specifically the learnability of IDDEAS at this stage. Our future research efforts will include iterations designated for service users testing and assessment of needs for optimal IDDEAS development. Despite qualitative research being highly dependent upon subjective interpretations and the relative competence of both the researcher and interviewee (12, 13), the combination of inductive and deductive qualitative categories within the QCA was found to be in line with findings from other studies (13). The use of COREQ and SRSS allowed for us to minimize possible bias and ensure adherence to previously validated qualitative standards. The IDDEAS project’s focus on formative usability assessments and prototype development allows for close collaboration with potential end-users in experimental settings, compensating for what could be deemed the limitation of qualitative interview inquiry. The mixed methods used in the usability evaluation will provide additional approaches to quantifying our findings, while also still allowing for efficiently identifying CAMHS clinicians’ current needs and how IDDEAS can meet those needs.

## Conclusion

Child and adolescent mental health services psychiatrists and psychologists shared the need for a completed IDDEAS clinical decision support system, especially if it is integrated within their clinical care processes; specifically, the electronic health record. Participants are eager to engage with the next phase, the dynamic high-fidelity prototype. Further usability assessments and identification of additional requirements for IDDEAS is necessary before feasibility testing and implementation. The findings from this study can help inform future IDDEAS development. A fully functioning, integrated version of IDDEAS has the potential to be an important contribution to support clinicians in the early identification of risks for mental disorders as well as full assessment and treatment of children and adolescents.

## Data availability statement

The raw data supporting the conclusions of this article will be made available by the authors, without undue reservation.

## Ethics statement

The studies involving human participants were reviewed and approved by the Regional Committee for Medical and Health Research Ethics, Southeast (REK Sør-Øst). The patients/participants provided their written informed consent to participate in this study.

## Author contributions

CC was responsible for conducting the data collection, data analysis, and formulation of the manuscript. NS and BL were responsible for providing the guidance and feedback throughout the development of the original manuscript and contributing with edits to the manuscript. All authors contributed with their suggestions throughout the development of the final manuscript and read and approved of the final manuscript.

## Footnotes

1
www.IDDEAS.no/
