# Supplementary material for: Usability of the IDDEAS prototype in child and adolescent mental health services: A qualitative study for clinical decision support system development
Source: Front Psychiatry. 2023 Feb 23;14:1033724. doi: 10.3389/fpsyt.2023.1033724 (PMC9997712; doi:10.3389/fpsyt.2023.1033724)
Supplement: Supplementary file 1 [file Table_1.DOCX]

Appendix i

**Interview Guide for qualitative IDDEAS prototype usability study**

*(*på norsk)*

1. Can you tell me about your overall experience of using IDDEAS?

*(Alt i alt, hva var din erfaring med å bruke IDDEAS?)*

1. Were there any specific aspects of IDDEAS that you liked? If so, could you explain specifically what/why?

*(Var det noe spesifikt du likte ved designet av IDDEAS? Hvis ja, kan du utdype hva/hvorfor?)*

1. Were there any specific aspects of IDDEAS that you did not like? If so, could you explain why and how you feel these could be improved?

*(Var det noe spesifikt du ikke likte ved designet av IDDEAS? Hvis ja, kan du utdype hva/hvorfor? og hvordan det kan forbedres?)*

1. In your opinion, how user-friendly was IDDEAS?

*(Hvor brukervennlig synes du IDDEAS var?)*

1. In your opinion, how aesthetically pleasing was IDDEAS?

*(Hvor estetisk tiltalende synes du IDDEAS var*?)

- 1. **For example**, was there anything missing from the screen display? If so, could you explain what you felt was missing from the display?

*(Følte du at det var noe som manglet på skjermen? Hvis ja, hva manglet?*

That is everything for today. Thank you very much for your help.

*Det er alt for i dag. Tusen, tusen takk for hjelpen din!*

*END*
