# Supplementary material for: Usability of the IDDEAS prototype in child and adolescent mental health services: A qualitative study for clinical decision support system development
Source: Front Psychiatry. 2023 Feb 23;14:1033724. doi: 10.3389/fpsyt.2023.1033724 (PMC9997712; doi:10.3389/fpsyt.2023.1033724)
Supplement: Supplementary file 2 [file Table_2.docx]

Appendix ii

**Part A: Version 1- Initial Version of the category system**

| **Main Category** | **Subcategory** |
| --- | --- |
| Category 1  Patient Case |  |
|  | Category 1.1  Referral Information |
|  | Category 1.2  Symptom History |
|  | Category 1.3  Diagnosis History |
|  | Category 1.4  Services Received History |
| Category 2  Software Functionality |  |
|  | Category 2.1  Validity of Information |
|  | Category 2.2  Guideline Content |
|  | Category 2.3  Approach to Support |
| Category 3  Usability |  |
|  | Category 3.1  Satisfaction |
|  | Category 3.2  Learnability |
|  | Category 3.3  Efficiency |
|  | Category 3.4  Memorability |
|  | Category 3.5  Errors |

**Part B: Category Revision**

- The following changes were made for Version 2:
- Refinement of patient case category
  - focus not on intentionally designed hypothetical patient cases, but rather on implications of the availability of information
- Refinement of subcategories under System functionalities; more directly reflect interview guide questions’ focus on IDDEAS specifically
  - Additional categories added for focus on EHR integration
- Refined usability subcategories slightly

**Part C: Version 2-Final version of the category system**

| **Main Category** | **Sub Category** |
| --- | --- |
| Category 1  Patient Information |  |
|  | Category 1.1  Patient Information & Referral Information Required |
|  | Category 1.2  Symptom History & Services Received |
|  | Category 1.3  Electronic Health Record Information Presentation |
|  |  |
| Category 2  Software Functionality & Content |  |
|  | Category 2.1  Validity of Content for CAMHS |
|  | Category 2.2  Aesthetic & design of user interface |
|  | Category 2.3  Approach to Support Layout |
|  |  |
| Category 3  Usability & Overall Experience |  |
|  | Category 3.1  Satisfaction |
|  | Category 3.2  Learnability |
|  | Category 3.3  Efficiency |
|  | Category 3.4  Memorability |
|  | Category 3.5  Errors |

**Part D: Coding Agenda & Description of Coding Tree**

| **Category**  **(C#)** | **Description** | **Example** | **Coding Rule** |
| --- | --- | --- | --- |
| C1- Patient Information  Interpretations of the information available about the patient and potential information requirements | | | |
| C1.1- Patient Information & Required Referral Information | What is the information presented about the patient and is there missing information required prior to continuing patient assessment? | *“Insufficient information for a referral-would be rejected in CAMHS”* | Mentions “missing/insufficient” patient information in reference to patient case interacting with. If about EHR display, code with C1.3 If functionality, C2. |
| C1.2- Symptom History and Services Received | The specific patient health record information available about previous care received, including the coordinated services within CAMHS. | “*Symptoms require understanding from additional services (PPT, Interviews w/parents)”; “Would want to follow up with PPT”* | Needs to mention additional services received or proposed as necessary |
| C1.3- Electronic Health Record Information Presentation | Reflections about presentation of the patient health information as simulated electronic health record system display | *“Is it going to be like where you write in—[…] if I have a patient, should I then write in all of the symptoms or the case?” “It is difficult to know how it would’ve been if I had my own case and had to put the symptoms into the system.”* | Needs to specify about EHR simulated display and interacting with the patient information as if it was a real patient EHR. If prototype functionality, move to C2. |
| C2- Software Functionality & Content | | | |
| C2.1-Validity of content for CAMHS | How the information provided in the guideline support reflects standard use of guidelines in CAMHS in Norway | *“It is good to see the information matches what I expect from ICD standards.”* | Must mention content of guideline in relation to CAMHS. If regarding functionality rather than wording of support, move to C2.3. |
| C2.2-Aesthetic & design of user interface | Visual appearance of the IDDEAS prototype interface | *“I think it was pleasing and not too messy.”* | If specifically mentions the aesthetics and design of visual layout. Focus on interface visual layout. If support function focused, move to C2.3. |
| C2.3- Approach to support layout | How the guideline support is presented and navigated through | *“I do not like the forced yes or no (decision tree format).” ; “following the guidelines helped me to structure the work”* | Must specifically mention interacting with the guideline/ provision of decision support. If content or display, move to C2.1 or C2.2. respectively. |
| C3-Usability and Overall Experience  Extent IDDEAS prototype is perceived as usable by CAMHS clinicians to achieve clinical goals | | | |
| C3.1-Satisfaction | Overall subjective perception of prototype at this stage to adequately provide support | *“I think it is easy to use […] but it is more about the usefulness. Up to this point it is just in this space it lacks something because of how it is. But it is user friendly.”* | Includes mention of meeting expectations currently and/or proposals for development to help achieve satisfaction. If specific to learnability/efficiency/memorability/errors, move to C3.2-C3.5 respectively. |
| C3.2- Learnability | Learning how to use the IDDEAS prototype and ability to follow guideline suggestions with successful performance of tasks | *"[...] it was of course quite new so a bit confusing but certain that after having used this twice or three or four times it would not be confusing anymore.”* | Includes mention of interactive system features learnability and ease of progression comfort using system. If specific to perceived satisfaction/efficiency/memorability/errors, move to C3.1, C3.3, C3.4, C3.5 respectively. |
| C3.3- Efficiency | How efficient was it to use IDDEAS for support? Forced to complete unnecessary tasks or unable to adequately present content relative to user. | *"I can see myself finding it more fun to do these evaluations, like it reminded me kind of some sort of game or it’s more pleasing to just look up in […] papers and ICD manuals.”* | Includes mention of necessary/unnecessary interactions with prototype guideline support impacting efficiency of clinical work. If specific to perceived satisfaction/learnability/memorability/errors, move to C3.1, C3.2 C3.4, C3.5. |
| C3.4- Memorability | Engagement of the system, including keeping track of components of clinical assessment | *“It was very intuitive and very easy[…] I think it was, you know normally I wouldn’t really think too much about such things and that’s probably a good thing, which means then it was probably fairly easy to move around inside.”* | Includes mention of the ability to remember clinical process while engaging with the prototype. If specific to satisfaction/learnability/efficiency/errors, move to C3.1, C3.2, C3.3, C3.5. |
| C3.5- Errors | Were there any errors encountered while using IDDEAS and how were any encountered errors acknowledged by system | *“Of course a glitch is making me skeptical. So the glitches should be repaired- but the logical side of it seems attractive to me.”* | Mention of errors encountered within the IDDEAS prototype guideline support and/or how apparent errors are acknowledged in interface. Mention of any speculated errors/proposition for future system error acknowledgement. If does not directly mention and specific to satisfaction/learnability/efficiency/memorability, move to C3.1, C3.2, C3.3, C3.4. |
